# Medium-Chain Fatty Acids and Breast Cancer Risk by Receptor and Pathological Subtypes

**DOI:** 10.3390/nu14245351

**Published:** 2022-12-16

**Authors:** Padmanabha Ganeshkodi Roopashree, Shilpa S. Shetty, Vijith Vittal Shetty, Suchetha Kumari Nalilu

**Affiliations:** 1 Central Research Laboratory, KS Hegde Medical Academy, Nitte (Deemed to be University), Mangalore 575018, India; 2 Department of Oncology, KS Hegde Medical Academy, Nitte (Deemed to be University), Mangalore 575018, India; 3 Department of Biochemistry, KS Hegde Medical Academy, Nitte (Deemed to Be University), Mangalore 575018, India

**Keywords:** breast cancer, medium-chain fatty acids, lauric acid, caprylic acid

## Abstract

**Introduction:** Medium-chain fatty acids contain 6–12 carbon atoms and are absorbed directly into the blood vessels, proceeding to the portal vein and, finally, to the liver, where they are immediately utilized for energy. We aimed to determine the medium-chain fatty acid levels in women with and without breast cancer. **Materials and Methods:** A total of 200 women (100 breast cancer subjects and 100 control subjects) were recruited for the study as per the inclusion and exclusion criteria. Blood samples were collected for biochemical estimations. Fatty acid methyl esters were isolated, and medium-chain fatty acid levels in plasma were analyzed using gas chromatography (GC-FID). Statistical analysis was performed using SPSS 20.0 software; *p* ≤ 0.05 was considered statistically significant. **Results:** The fatty acid analysis revealed a significant decrease in the levels of caprylic acid (C:8) and lauric acid (C:12) and a significant increase in the level of capric acid (C:10) in the breast cancer subjects when compared to the control group. The level of caproic acid (C:6) was not significantly increased in the breast cancer subjects. In particular, the HER2- and ER-positive breast cancer subjects showed a decrease in their caprylic acid and lauric acid levels compared to other receptors. **Conclusions:** The results of the current study imply that lower levels of caprylic and lauric acid may be associated with a higher risk of breast cancer. The relevance of medium-chain fatty acids for preventive and therapeutic interventions will be amplified by further research on the possibility that alteration in a patient’s medium-chain fatty acid composition may mechanistically contribute to disease progression or breast cancer risk.

## 1. Introduction

Breast cancer is the major cause of cancer-related death in women. In India, in the year 2018, 162,468 women were newly diagnosed with breast cancer and 87,090 women died of breast cancer [1]. It is indisputable that the existing chemotherapy is effective in cancer treatment, though the presence of undesirable adverse effects has activated a demand for new therapeutic agents. Breast cancer risk factors have been recognized, including hereditary inheritance, environmental exposure, infection, reproductive characteristics, and diet. The nutritional risk factors of breast cancer include dietary fats [2], even though the epidemiological evidence is still ambiguous. This controversy exists partially because body fat is usually studied according to the total fat content, the type of fatty acid (saturated, monounsaturated, or polyunsaturated), or in terms of origin. Therefore, even though certain fatty acids are thought to have a significant impact on a variety of biological processes, including tumor development and progression, little attention has been paid to them.

Medium-chain fatty acids (MCFAs) are saturated fatty acids with 6 to 12 carbon atoms. Coconut oil (CO) and palm kernels are some natural sources. Increased levels of long-chain fatty acids (LCFA) are also related to an increased risk of breast cancer [3]. Contrary to LCFAs, MCFAs must be combined into chylomicrons before entering the liver. However, dietary medium-chain fatty acids are quickly absorbed in the gastrointestinal tract and are transported into the bloodstream through direct contact with albumin [4,5] via the portal vein; once there, they reach the liver and are metabolized through β-oxidation in mitochondria [6]. Caproic acid (C:6), caprylic acid (C:8), capric acid (C:10), and lauric acid (C:12) are the most common medium-chain fatty acids. They have proven anticancer effects on human breast, skin, and colorectal cancer cells invitro [6]. Coconut oil, a source of MCFA’s is a credible nutraceutical for cancer prevention [7]. A cohort study has revealed that MCFAs are valuable early diagnostic biomarkers of colorectal cancer [8]. To potentiate our understanding of the possible relationship between MCFAs and breast cancer, this case–control study was undertaken on a relatively stable and homogeneous population to determine the level of specific MCFAs in breast cancer subjects and controls and to assess the possible association between specific MCFAs and breast cancer risk.

## 2. Materials and Methods

The study was approved by the Central Ethics Committee of Nitte (Deemed to be a University). A total of 100 histopathologically proven breast cancer subjects and 100 control subjects attending the OPD for general health check-ups between the age of 25–60 were recruited for the study after obtaining informed consent. Demographic data such as age, BMI (Body Mass Index), diet, menopausal status, and first-degree family history with biochemical parameters like Hb (Hemoglobin), RBS (Random blood sugar), platelet count, blood urea, creatinine concentration, ALP (Alkaline phosphatase), AST (Aspartate aminotransferase), ALT (Alanine transaminase), Na^+^, K^+^, Cl^−^, total bilirubin, albumin, globulins, A/G (Albumin/globulin) ratio, and total protein were noted. Clinical characteristics such as TNM (Tumor Node Metastases) stage, tumor size, receptor status, grade, and histological type were noted in breast cancer subjects. A total of 3 mL of blood was collected for from each subjects.

### 2.1. Selection of Subjects

The study group included 100 women as per the inclusion and exclusion criteria. Subjects diagnosed with breast cancer proven through pathology reports without any treatment were included in the study. Subjects who had breast implants or any malignancies were excluded from the study. We recruited 100 age-matched control women who attended the oncology OPD and not undergone breast-conserving surgery/not reported breast cancer at the time of enrollment. Control subjects, pregnant women, and any other benign proliferations were excluded from the study. A total of 3 mL of the blood was collected from the recruited subjects, plasma samples and stored at −20 °C and until further analysis.

### 2.2. Estimation of Lipid Profiles

TC (Total Cholesterol), TG (Triglycerides), and HDL-C (High-density lipoprotein cholesterol) levels were analyzed using commercially available kits (Liqui CHEKTM AGAPPE). Friedewald formula was used for LDL-C (Low-density lipoprotein cholesterol) and VLDL-C (Very low-density lipoprotein cholesterol) calculation [9]. The formation of malondialdehyde (MDA) was estimated using a standardized protocol by Buege, J. A et al. [10].

### 2.3. Medium-Chain Fatty Acid Analysis

Lipid transesterification of stored plasma to fatty acid methyl esters (FAMEs) was performed according to the modified protocol of Metcalfe et al. [11]. Fatty acid levels were determined in the presence of internal standard (1 mg/mL methyl heptadecanoate-C17:0, Sigma Aldrich). Extraction of the total plasma medium-chain fatty acid was performed via the hydrolysis of esters and then derivatization of esters under alkaline conditions in 14% boron trifluoride-methanol for 5 min at 100 °C to form FAMEs. Then, these fatty acids were measured using gas chromatography (GC-FID). Extracted FAMEs were analyzed on a 7820A Agilent GC-FID (flame ionization detector) with J and W DB-23 high-quality columns. Individual medium-chain fatty acids were identified by comparing their elution times with relative medium-chain fatty acid standards. Fatty acids were calculated according to their comparative abundance with respect to the internal standard added. The quantity of individual medium-chain fatty acid was calculated as the percentage of the total medium-chain fatty acid concentration within each sample.

## 3. Statistical Analysis

Statistical analysis of the obtained results was performed using GraphPad Prism, Version 8.0.2 (GraphPad Software, Inc., La Jolla, CA, USA). Categorical variables were analysed using a chi-square test. Student’s *t*-test was used to compare the two groups. The Mann–Whitney U test was used for non-parametric variables. To correlate the non-parametric variables with each other for all subjects, the Spearman’s correlation coefficient test was used. All statistical tests were two-sided, and *p* < 0.05 was considered significant.

## 4. Results

### 4.1. General Characteristics of the Study Population

A total of 200 participants were included in this study. Of them, 100 were control subjects and 100 were breast cancer subjects. The demographic characteristics are summarized in Table 1. The mean age (±SD) was 46.731 ± 10.846 years for the control subjects and 50.04 ± 10.611 years for the breast cancer subjects, and the age distribution of the control subjects and breast cancer subjects was similar. Out of 100 control subjects, 52% were pre-menopausal and 48% were post-menopausal, whereas in the 100 breast cancer subjects, 31% were pre-menopausal and 69% were post-menopausal. Out of the 100 control subjects, 3% had a BMI of <18.5, 70% had BMI of 18.5–24.9, and 27% had a BMI of ≥25. Whereas in the breast cancer subjects, 2% had a BMI of <18.5, 58% had a BMI of 18.5–24.9, and 40% had a BMI of ≥25. The mean BMI and age showed a significant difference between the breast cancer subjects and the control subjects. The family history of the breast cancer in the first-degree relatives among the cases and controls indicated that the cases were likely to have a higher proportion of first-degree relatives (mother, sisters, and daughters) with breast cancer (<0.040). History regarding menopausal status and diet did not exhibit a significant case–control difference in the present study.

Among the 100 breast cancer subjects, 63 had invasive ductal carcinoma and 37 had invasive lobular carcinoma. Concerning the clinicopathological differences among the individuals in the two types of breast cancer groups, there was no significant difference observed with TNM stage, tumor size, lymph node status, and receptor status of breast cancer, but a significant difference was observed concerning grade (*p* < 0.027). The clinicopathological characteristics are summarized in Table 2.

### 4.2. Comparison of Biochemical Parameters in the Study Population

The random blood glucose level was significantly higher in the breast cancer subjects than in the control group. The mean Hb level was significantly lower in the breast cancer subjects than in the control group. The levels of TC, TG, HDL-C, LDL-C, VLDL-C, and MDA were also examined and are shown in Table 3. The levels of TC, TG, VLDL-C, and MDA in the breast cancer subjects were significantly increased when compared to the control group. Whereas the HDL-C levels were significantly decreased in the breast cancer subjects. The mean values regarding the ALT, blood urea, creatinine, albumin, and globulin levels were significantly increased in the breast cancer group compared to the control group.

In addition, the percentages of medium-chain fatty acids, i.e., caproic acid, caprylic acid, capric acid, and lauric acid, in each group are represented in a box plot (Table 3). The results showed the levels of MCFAs in the control and breast cancer subjects, while the most statistically significant decrease in caprylic acid and lauric acid was seen in the breast cancer subjects. In particular, the levels of capric acid were statistically significantly higher in the breast cancer subjects than in the control group. The levels of caproic acid were higher in the breast cancer subjects than in the control group, but no significant difference was observed.

### 4.3. Age-Wise Distribution of MCFA Levels in the Study Population

**Figure 1:**
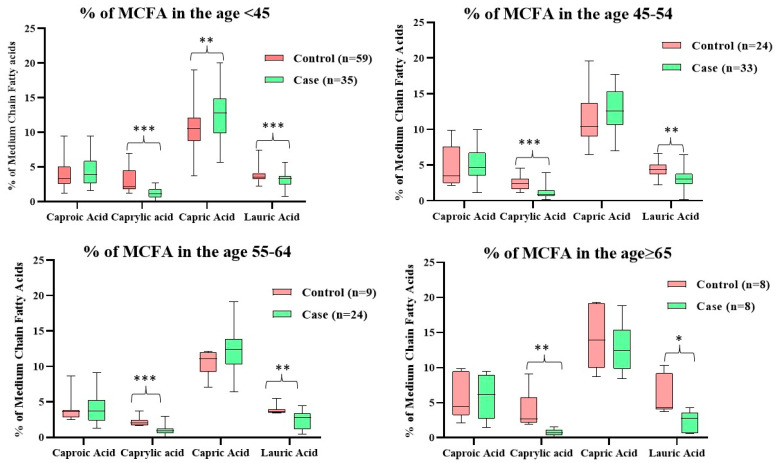
Age-wise distribution of MCFA levels in control and breast cancer subjects. The amount of each MCFA was calculated as a % of total MCFAs. *p*-value was calculated using Mann–Whitney U test for non-parametric variables. Data are shown as median (interquartile range). *** *p* < 0.0001, ** *p* < 0.001, and * *p* < 0.05. Abbreviations: MCFA—Medium-chain fatty acid.

In the 100 breast cancer subjects, 35% of the subjects were <45 years old, 33% of the subjects were 45–54 years old, 24% of the subjects were 55–64 years old, and 8% of the subjects were ≥65 years old. Whereas in the 100 control subjects, 59% of the subjects were <45 years old, 24% of the subjects were 45–54 years old, 9% of the subjects were 55–64 years old, and 8% of the subjects were ≥65 years old. The box plot shows lower levels of caprylic and lauric acids in the different age groups of the breast cancer subjects than in the control group, which were statistically significant. The distribution of MCFAs in each age group of the study subjects is reported in the box plot (Figure 1). In the entire <45-year-old demographic, the level of capric acid was significantly higher in the breast cancer subjects compared to the control group. However, there were no significant differences observed in the other age groups of the study subjects.

### 4.4. Comparison of MCFA Levels Regarding Histological Types, TNM Stage, and Grade of Breast Cancer Subjects

**Figure 2:**
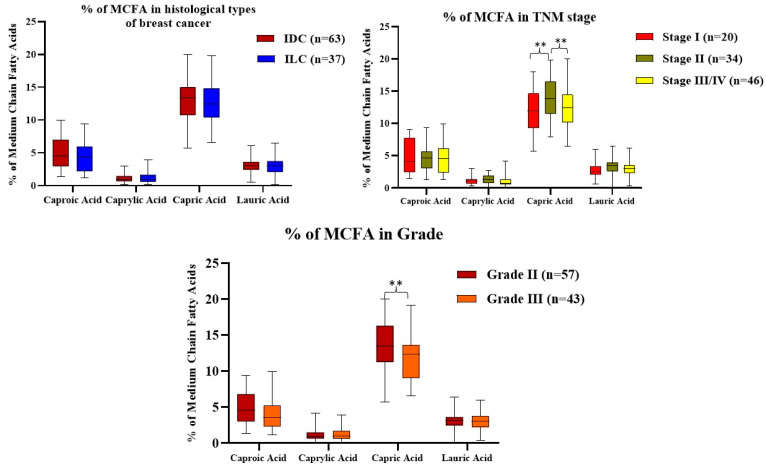
Comparison of MCFA levels in terms of histological types, TNM stage, and grades of breast cancer subjects. The amount of each MCFA was calculated as a % of total MCFAs. *p*-value was calculated using the Mann–Whitney U test for non-parametric variables. Data are shown as median (interquartile range). ** *p* < 0.001. Abbreviations: TNM—Tumor Node Metastases; Tumor IDC—Invasive ductal carcinoma; ILC—Invasive lobular carcinoma; MCFA—Medium-chain fatty acid.

A comparison of the MCFAs with respect to the histological types, TNM stage, and grade of the breast cancer subjects is shown in Figure 2. Regarding tumor histology, 63% of the breast cancer subjects had invasive ductal carcinoma and 37% of the breast cancer subjects had invasive lobular carcinoma. There were no significant differences observed regarding the histological types of the breast cancer subjects. Regarding the TNM stage, 20% of the breast cancer subjects corresponded to stage I, 34% of the breast cancer subjects corresponded to stage II, and 46% of the breast cancer subjects corresponded to stage III/IV. The level of capric acid was significantly increased in the stage II tumor compared to the stage I tumor breast cancer subjects, whereas the level of capric acid was significantly decreased in the stage III/IV tumor compared to the stage II tumor subjects. However, there was no significant difference observed with respect to the other MCFA levels. Concerning grade, 57% of the breast cancer subjects corresponded to grade I, and 43% of the breast cancer subjects corresponded to grade II. The level of capric acid was significantly decreased in grade III compared to grade II, but no significant difference was observed in the other MCFA levels.

### 4.5. Distribution of MCFAs Levels with Respect to the Receptor Status of the Breast Cancer Subjects

**Figure 3:**
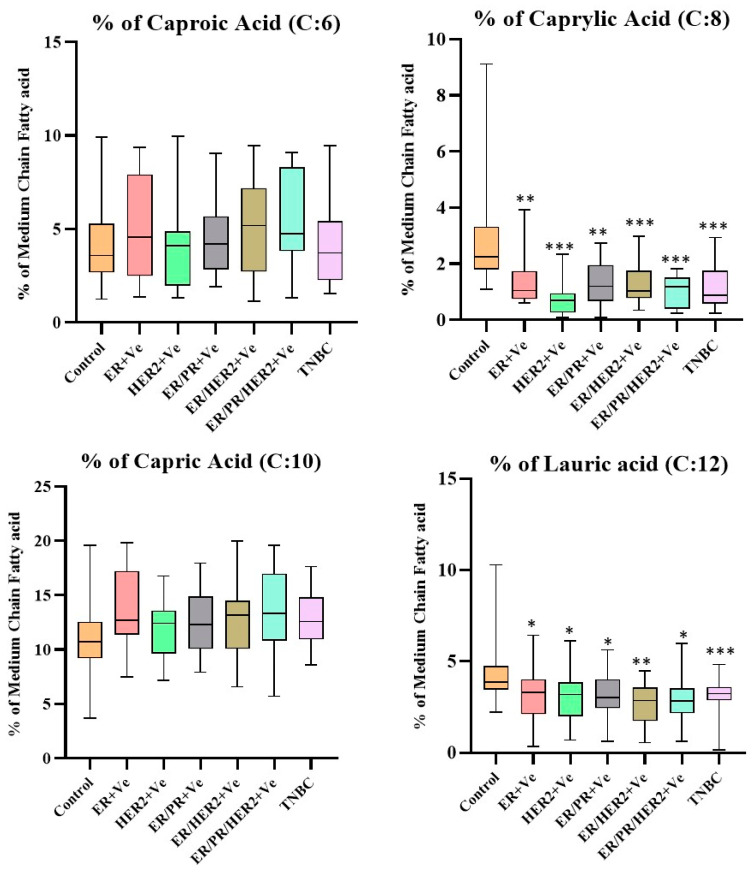
Tukey boxplots showing the distributions of the levels of each MCFA corresponding to various receptor statuses of breast cancer subjects. The quantity of each MCFA was calculated as a % of total MCFAs. *p* value was calculated using Mann–Whitney U test for non-parametric variables. ER + Ve (*n* = 17), HER2 + Ve (*n* = 18), ER/PR + Ve (*n* = 16), ER/HER2 + Ve (*n* = 18), ER/PR/HER2 + Ve (*n* = 15), and TNBC (*n* = 16). *** *p* < 0.0001, ** *p* < 0.001, and * *p* < 0.05. Abbreviations: ER—Estrogen Receptor; HER2—Human Epidermal Growth Factor Receptor 2; PR—Progesterone Receptor; TNBC—Triple-Negative Breast Cancer; MCFA—Medium-chain fatty acid.

According to receptor status, the differences in the MCFA levels in the breast cancer subjects became more evident (Figure 3). Of the 100 breast cancer subjects, 17% of the breast cancer subjects were ER-positive, 18% of the breast cancer subjects were HER2-positive, 16% of the breast cancer subjects were ER/PR-positive, 18% of the breast cancer subjects were ER/HER2-positive, 15% of the breast cancer subjects were ER/PR/HER2-positive, and 16% of the breast cancer subjects had TNBC. Both HER2-positive and ER/HER2-positive breast cancer were frequent findings in our study compared to other receptor statuses. The corresponding box plot shows significant decreases in the levels of caprylic and lauric acids at various receptor statuses compared to the control group. In particular, the level of caprylic acid was significantly lower in the HER2-positive breast cancer subjects than in the other receptor statuses of the breast cancer subjects. The level of lauric acid was lower and statistically significant in the ER/HER2-positive breast cancer subjects compared to the other receptor status. Caproic and capric acids did not show any statistically significant differences with receptor status in breast cancer subjects.

### 4.6. Correlation between Hematological Parameters, Lipid Profile, Kidney Function Test, Liver Function Test, and Medium-Chain Fatty Acids in Breast Cancer

A significant positive correlation was observed between caproic acid and TC, whereas a significant negative correlation was seen between ALT, the total bilirubin content, and the A/G ratio. Caprylic acid showed a significant negative correlation with TC, AST, and globulin compared to the other parameters. Capric acid showed a significant negative correlation with ALT and AST. Lauric acid showed a significantly negative correlation with TC, LDL-C, VLDL-C, AST, and globulin (Supplementary Table S1).

## 5. Discussion

The complex variations in the metabolism of carbohydrates, proteins, and lipids are essential for tumor cells’ growth and proliferation. In many epidemiological studies, increased dietary fat consumption is positively associated with breast cancer. In the present study, significantly high levels of TC, TG, and VLDL-C were observed, but the level of HDL-C was significantly decreased in breast cancer subjects. More lipids are needed to improve signaling and the resistance to apoptosis in rapidly multiplying cancer cells [12]. An increased plasma LDL-C concentration increases exposure to oxidation, causing higher lipid peroxidation in breast cancer subjects [13]. In this study, the level of VLDL-C was significantly increased in the breast cancer subjects (Table 3).

High levels of SGOT (Serum glutamic-oxaloacetic transaminase) and SGPT (Serum glutamic-pyruvic transaminase) suggest that liver and kidney function impairment might be triggered by tumor invasion [14]. This study observed a significantly decreased albumin level in breast cancer subjects. This reduced albumin level corresponds to the poor survival of breast cancer subjects [15,16,17]. An elevated creatinine level causes kidney impairment [18]. This study found a statistically significant realtion to creatinine level. Glucose plays a vital role in breast cancer therapy. Some studies have shown that hematological and solid tumor hyperglycemia is linked with increased toxicity [19]. In this study, significant changes were noted concerning random blood glucose levels in breast cancer subjects. Based on the clinical findings concerning the subjects diagnosed with breast cancer, our investigations showed that the levels of urea were significantly lower compared to the control group. Decreased urea levels might suggest a link between the dysregulation of protein catabolic processes and the aggressive behavior of cancer cells [20]. A common complication in breast cancer subjects is anemia. This study observed significantly low hemoglobin levels in breast cancer subjects (Table 3). Higher levels of inflammatory markers, IL-6, leptin, hepcidin, ferritin, and ROS all contribute to anemia in cancer subjects.

The traditional sources of fatty acids include diet, circulation from adipose tissue, and surplus carbohydrates the liver that turn into fat. Most human diets contain various saturated fatty acids of different carbon chain lengths. MCFAs are saturated fatty acids with 6–12 carbon atoms that are more quickly taken from the intestine to the liver via the portal vein and immediately used for energy. Naturally, medium-chain fatty acids are found in coconut oil, palm kernel oil, and in milk fat [21,22,23]. Though no association between medium-chain fatty acids and gut microbes has been documented, Caprylate (C8), one of the MCFAs, has been reported to be produced by specific yeast strains [24]. Clostridium kluyveri [25,26,27], a bacterial strain found in the rumen intestine [28,29], can produce MCFAs for industrial uses. However, there are few findings on MCFAs generated from gut bacteria in non-rumen animals. It is interesting to note that MCFAs have anti-bacterial and anti-fungal effects on specific bacterial strains [30,31]. Less than 2% of dietary energy is typically contributed by MCFAs in the modern human diet [32]. While PUFAs are generally known as anticancer dietary components, MCFAs have also been described to have a therapeutic role [33,34]. In the present study, we analyzed the plasma medium-chain fatty acid levels in control and breast cancer subjects. Our results showed a significant decrease in caprylic acid and lauric acid levels in the breast cancer subjects compared to the control group, except for caproic and capric acid (Table 3). In this study, the plasma medium-chain fatty acid levels were measured because plasma fatty acid levels depend on dietary intake, and these sources can provide a more objective measure of fatty acid levels than estimates based on dietary intake [35].

Among the saturated medium-chain fatty acids, the caprylic acid concentration was higher in the control group and lower in the breast cancer subjects. Caprylic acid is enriched in coconut and goat’s milk. Narayanan et al. [6] reported that caprylic acid inhibited the viability of skin, colorectal, and breast cancer cells and downregulated the expression of genes such as CDK2, CDK4, CCNA2, and CCND1, which are mainly involved in progression and cell cycle division in colon cancer cells. A mechanism connected to ABCA1 and the p-JAK2/pSTAT3 signaling pathway suggests that caprylic acid may be crucial for lipid metabolism and the inflammatory response [36]. Yamasaki et al. [37] suggested that octanoic acid inhibits bladder cancer cell proliferation but does not reduce cell migration and invasion. Studies on the association between the levels of caprylic acid and the prognosis of breast cancer are limited. Jansen et al. [38] indicated that consuming full-fat products including saturated fatty acids such as octanoic acid increases the risk of pancreatic cancer dose-dependently. According to Cuizhe Wang et al. [39], caprylic acid (C8:0) enhances COX2 and PGE2 expression in the bone marrow cavity, increases adipocyte growth and proliferation, and causes bone metastases of prostate cancer. According to a study by Iemoto et al. [40], people with colorectal cancer who had lower levels of serum caprylic acid (C8:0) had a better prognosis than those who had greater levels of caprylic acid. However, to the best of our knowledge, no studies assessing the effect of caprylic acid on the risk of breast cancer have been detailed; hence, more research is required.

In this study, we found that the level of lauric acid in the breast cancer subjects was significantly lower than in the control group. The major source of lauric acid is coconut oil and palm kernel oil. Lauric acid promotes cell death, which is assisted through the activation of EGFR and the Rho-associated kinase pathway, according to Lappano et al. [41]. According to research by Sheela et al. [7], lauric acid significantly inhibits the growth of human hepatocellular carcinoma and murine macrophage cells. Lauric acid and increased intracellular reactive oxygen species with a corresponding decrease in the intracellular reduced glutathione levels, have been demonstrated to cause apoptotic alterations and cell cycle arrest in the G0/G1 and G2/M phases.

According to numerous studies, postmenopausal older women in industrialized countries are more likely to acquire breast cancer than younger premenopausal women. In our study, the age-wise distribution of caprylic and lauric acids was significantly lower in the breast cancer subjects than in the control group (Figure 1). The younger population and unique demographics of developing countries may have a significant impact on these findings [1,42].

In this study, Figure 2 depicted the levels of medium-chain fatty acids by grade. Out of the 100 breast cancer individuals, 43% of those with grade III and 57% of those with grade II had the disease. When compared to other saturated medium-chain fatty acids, grade III had much less capric acid than grade II. In the stage II breast cancer patients, compared to stage I, the capric acid levels were higher; however, in the stage III/IV breast cancer patients, compared to stage II, the capric acid levels were lower. The most prevalent finding in our investigation related to grade 2 and stage 2 or 3 tumors, which was comparable to the results from a study of symptomatic cases conducted in the UK [43].

In the present study, we found that the caprylic and lauric acid levels were significantly lower in all breast cancer subjects receptor subtypes compared to control subjects (Figure 3). Based on the receptor status of the breast cancer subjects, caprylic acid was lower in the HER2-positive breast tumors, whereas lauric acid was lower in the ER/HER2-positive breast tumors compared to the other receptor groups. These findings suggested that out of the 100 breast cancer subjects, 18% had HER2-positive and ER/HER2-positive tumors. The ER status in the breast tumor cells was assessed in a study by Mirtavoos-Mahyari et al. (2014) to determine how it affected the activation of the tyrosine kinase human epidermal growth factor receptor 2 (HER2). Consequently, 31% of breast cancer participants and 67% overall had HER2 + tumors, according to the study [44]. Similarly, Rodrigue et al. (2014) found that ER + tumors were present in 61% of their study’s breast cancer participants [45]. These studies linked increased steroid hormone responsiveness, a higher BMI, and increased body fat to hormone receptor-positive breast cancer.

The caproic acid levels showed a weakly positive link with total cholesterol and a weakly negative correlation with ALT, total bilirubin, and the A/G ratio in the breast cancer individuals. Between total cholesterol, AST, and globulin, there was a weakly negative association with the caprylic acid levels. While the lauric acid levels showed a weakly negative association with triglycerides, LDL-C, VLDL-C, AST, and globulin, capric acid levels showed a weakly negative correlation with AST and ALT (Supplementary Table S1).

There is a knowledge gap concerning the mechanism of action of medium-chain fatty acids on the oncogenic signal transduction pathway, and also clinical studies are limited. Identifying how medium-chain fatty acids are associated with the oncogenic signal transduction regulation and clinical presentation of breast cancer will be key in elucidating the mechanism behind this disease. Our study is vital in demonstrating the importance of medium chain fatty acid in cancer management and prevention.

## 6. Conclusions

There are considerable barriers o improving the prognosis of breast cancer subjects due to the lack of diagnostic technologies that are characterized by substantial patient compliance and good clinical applicability. Here, we have described the levels of different MCFAs in human samples. Among the medium-chain fatty acids investigated, the levels of caprylic and lauric acid were decreased in the subjects with breast cancer. Our findings imply that increasing the intake of caprylic and lauric acid while lowering the intake of caproic and capric acids may be an effective strategy for preventing breast cancer. To fully comprehend the impact of these medium-chain fatty acids on the development of breast cancer, more in vitro and in vivo investigations are required. The possible mechanisms of MCFA’s effects must be determined, to further understand and enhance the use of MCFAs as a complementary breast cancer treatment and their effectiveness across breast cancer receptor and pathological subtypes. Further investigation would increase the knowledge and understanding of fatty acids and breast cancer, including the impact of MCFAs on the overexpressing receptor subtypes. Future research in this field should focus on the effect of early MCFA exposure on long-term breast cancer risk.

## Figures and Tables

**Table 1:** Demographic characteristics of the study population.

Variables	Control (*n* = 100)	Case (*n* = 100)	*p* Value
**Age**			
Total	46.731 ± 10.846	50.04 ± 10.611	<0.04 *
<45	59	35	0.072
45–54	24	33	
55–64	9	24	
≥65	8	8	
**BMI**			
Total	21.232 ± 2.870	22.117 ± 3.448	<0.001 **
<18.5	3	2	0.050
18.5–24.9	70	58	
≥25	27	40	
**Menopausal status (%)**			
Pre-menopausal	52	31	0.072
Post-menopausal	48	69	
**First-degree family history (%)**			
Yes	2	11	<0.040 *
No	98	89	
**Diet (%)**			
Mixed	87	85	0.833
Vegetarian	13	15	

Age and BMI is represented as mean ± SD. Subgroups of Age and BMI, Menopausal status, First-degree family history, and Diet variables are given in percentages. *p* value was calculated using the Student’s *t*-test for parametric variables. ** *p* < 0.001; * *p* < 0.05. Categorical variables was tested using the chi-square test.

**Table 2:** Clinical characteristics of women with breast cancer.

Tumor Characteristics	Invasive Ductal Carcinoma *n* (%) (*n* = 63)	Invasive Lobular Carcinoma *n* (%) (*n* = 37)	*p* Value
**TNM Stage**			
I	11 (18)	9 (24)	0.477
II	24 (38)	10 (27)	
III/IV	28 (44)	18 (49)	
**Tumor size (cm)**			
<2.0	16 (25)	16 (43)	0.105
2.0–4.9	20 (32)	6 (16)	
≥5.0	27 (43)	15 (41)	
**Lymph Node status**			
Positive	42 (67)	25 (68)	0.553
Negative	21 (33)	12 (32)	
**Receptor status**			
ER + Ve	6 (10)	11 (30)	0.144
HER2 + Ve	14 (22)	4 (11)	
ER/PR + Ve	11 (18)	5 (13)	
ER/HER2 + Ve	11 (17)	7 (19)	
ER/PR/HER2 + Ve	11 (17)	4 (11)	
TNBC	10 (16)	6 (16)	
**Grade**			
2	41 (65)	16 (43)	<0.027 *
4	22 (35)	21 (57)	

The variables—TNM stage, tumor size, lymph node status, receptor status, and grade—are presented as percentages. For categorical variables, the statistical significance of the two groups was tested using a chi-square test. * *p* < 0.05 was considered statistically significant. Abbreviations: TNM—Tumor Node Metastases; ER—Estrogen Receptor; HER2—Human Epidermal Growth Factor Receptor 2; PR—Progesterone Receptor; TNBC—Triple-Negative Breast Cancer.

**Table 3:** Comparison of the biochemical parameters in subjects with and without breast cancer.

Parameters	Control (*n* = 100)	Breast Cancer (*n* = 100)	*p* Value
RBS (mg/dL)	105.332 ± 7.695	136.600 ± 72.074	<0.001 **
**Hematological Parameters**			
Hb (g/dL)	13.273 ± 0.849	10.894 ± 1.375	<0.001 **
Platelet count (10^3^/µL)	336.549 ± 107.376	252.360 ± 93.006	0.061
**Lipid Profiles**			
TC (mg/dL)	157.430 ± 27.185	197.180 ± 33.065	<0.021 *
TG (mg/dL)	123.191 ± 34.028	152.228 ± 53.672	<0.001 **
HDL-C (mg/dL)	59.075 ± 19.592	53.766 ± 15.669	<0.017 *
LDL-C (mg/dL)	60.279 ± 19.865	65.568 ± 19.777	0.888
VLDL-C (mg/dL)	24.546 ± 7.046	29.977 ± 11.191	<0.001 **
**Lipid Peroxidation**			
MDA (µM/L)	3.867 ± 1.882	4.137 ± 0.441	<0.04 *
**Liver Function tests**			
ALP (IU/L)	107.381 ± 30.061	107.520 ± 29.892	0.586
AST (IU/L)	24.448 ± 16.435	26.686 ± 9.913	0.571
ALT (IU/L)	17.553 ± 9.814	33.087 ± 15.573	<0.001 **
Total Bilirubin (mg/dL)	0.464 ± 0.166	1.257 ± 0.520	0.137
**Kidney Function tests**			
Blood Urea (mg/dL)	18.314 ± 6.224	14.539 ± 4.752	<0.001 **
Serum Creatinine (mg/dL)	0.707 ± 0.140	1.607 ± 8.526	<0.032 *
Albumin (g/dL)	3.958 ± 0.856	4.290 ± 0.635	<0.011 *
Globulin (g/dL)	2.840 ± 0.280	3.167 ± 0.434	<0.001 **
A/G Ratio	1.574 ± 0.680	1.676 ± 0.733	0.306
Total Protein (mg/dL)	7.256 ± 0.946	7.394 ± 0.860	0.086
Na^+^ (mmol/L)	137.442 ± 15.349	139.442 ± 6.509	0.796
K^+^ (mmol/L)	4.180 ± 0.485	4.333 ± 0.694	<0.013 *
Cl^−^ (mmol/L)	99.604 ± 11.432	102.323 ± 4.176	0.937
**Medium-chain fatty acids**			
Caproic Acid (%, C:6)	3.594 (2.684–5.309)	4.516 (2.671–6.529)	0.302
Caprylic Acid (%, C:8)	2.256 (1.794–3.318)	0.902 (0.624–1.547)	<0.001 ^#^
Capric Acid (%, C:10)	10.709 (9.198–12.544)	12.559 (10.393–14.956)	<0.002 ^#^
Lauric Acid (%, C:12)	3.882 (3.470–4.761)	3.083 (2.366–3.700)	<0.001 ^#^

*p* value ≤ 0.05 was considered statistically significant. * Student’s *t*-test was used for parametric variables; the data are represented as mean ± SD. ^#^ Mann–Whitney U test was used for non-parametric variables; data are shown as medians (interquartile range). ** *p* < 0.001 and * *p* < 0.05. Abbreviations: RBS—Random blood sugar, Hb—Hemoglobin, MDA—Malondialdehyde, TC—Total Cholesterol, TG—Triglycerides, HDL-C—High-density lipoprotein cholesterol, LDL—Low-density lipoprotein cholesterol, VLDL-C—Very low-density lipoprotein cholesterol, ALP—Alkaline phosphatase, AST—Aspartate aminotransferase, ALT—Alanine transaminase, and A/G (Albumin/globulin) ratio.

## Data Availability

The data are not publicly available.

## Supplementary Materials

The following supporting information can be downloaded at: https://www.mdpi.com/article/10.3390/nu14245351/s1, Table S1. Correlation between lipid profiles, liver function test, hematological parameters, kidney function test, and medium chain fatty acids in breast cancer.
