# Supplementary material for: Medium-Chain Fatty Acids and Breast Cancer Risk by Receptor and Pathological Subtypes
Source: Nutrients. 2022 Dec 16;14(24):5351. doi: 10.3390/nu14245351 (PMC9781514; doi:10.3390/nu14245351)
Supplement: Supplementary file 1 [file nutrients-14-05351-s001.zip › nutrients-2042405-supplementary.pdf]

Table S1. Correlation between lipid profiles, liver function test, hematological parameters, kidney function test, and medium chain fatty acids in breast cancer.

		Caproic Acid (%)	Caprylic Acid (%)	Capric Acid (%)	Lauric Acid (%)
**Hb (g/dl)**	Correlation	-0.092	-0.138	-0.094	-0.103
	p- Value	0.358	0.171	0.350	0.310
**Platelet count (10^3 *µl)**	Correlation	0.058	-0.034	0.133	-0.071
	p- Value	0.568	0.738	0.188	0.485
**RBS (mg/dl)**	Correlation	0.091	-0.022	-0.012	0.089
	p- Value	0.370	0.828	0.907	0.378
**TC (mg/dl)**	Correlation	0.233	-0.291	0.136	-0.036
	p- Value	<0.020*	<0.003*	0.177	0.725
**TG (mg/dl)**	Correlation	0.005	-0.009	0.043	-0.212
	p- Value	0.963	0.929	0.668	<0.034*
**HDL-C (mg/dl)**	Correlation	0.137	0.050	0.079	0.165
	p- Value	0.174	0.624	0.436	0.102
**LDL-C (mg/dl)**	Correlation	0.170	-0.168	0.137	-0.207
	p- Value	0.092	0.094	0.173	<0.039*
**VLDL-C (mg/dl)**	Correlation	0.025	-0.059	-0.039	-0.253
	p- Value	0.801	0.558	0.701	<0.011*
**MDA (µM/L)**	Correlation	-0.037	-0.119	0.049	-0.041
	p- Value	0.716	0.240	0.626	0.685
**Blood Urea (mg/dl)**	Correlation	-0.104	-0.092	-0.010	-0.077
	p- Value	0.304	0.363	0.919	0.446
**Serum Creatinine (mg/dl)**	Correlation	0.035	-0.020	-0.161	0.037
	p- Value	0.733	0.845	0.111	0.717
**ALP (IU/L)**	Correlation	-0.043	-0.163	-0.177	-0.161
	p- Value	0.668	0.104	0.078	0.111
**AST (IU/L)**	Correlation	-0.193	-0.251	-0.292	-0.257
	p- Value	0.054	<0.012*	<0.003*	<0.010*
**ALT (IU/L)**	Correlation	-0.224	-0.179	-0.226	-0.109
	p- Value	<0.015*	0.075	<0.024*	0.282
**Na + (mmol/L)**	Correlation	0.060	-0.020	0.089	0.123
	p- Value	0.556	0.842	0.380	0.222
**K+ (mmol/L)**	Correlation	-0.158	0.034	-0.142	-0.050
	p- Value	0.116	0.738	0.160	0.618
**Cl - (mmol/L)**	Correlation	-0.186	0.079	-0.140	-0.099
	p- Value	0.064	0.435	0.165	-0.070
**Total Bilirubin (mg/dl)**	Correlation	-0.223	-0.131	-0.143	-0.015
	p- Value	<0.026*	0.194	0.155	0.491
**Albumin (g/dl)**	Correlation	-0.021	0.136	0.145	0.013
	p- Value	0.840	0.178	0.150	0.900
**Globulin (g/dl)**	Correlation	0.076	-0.207	0.045	-0.198
	p- Value	0.451	<0.039*	0.653	<0.048*
**A/G Ratio**	Correlation	-0.217	0.085	-0.151	-0.055
	p- Value	<0.030*	0.399	0.135	0.587
**Total Protein (mg/dl)**	Correlation	-0.002	-0.056	-0.049	-0.009
	p- Value	0.985	0.579	0.629	0.928

***P value≤0.05 was considered statistically significant.** Abbreviations: RBS - Random blood sugar, Hb - Hemoglobin, MDA – Malondialdehyde, TC - Total Cholesterol, TG - Triglycerides, HDL-C - High-density lipoprotein cholesterol, LDL- Low-density lipoprotein cholesterol, and VLDL-C - Very low-density lipoprotein cholesterol, ALP - Alkaline phosphatase, AST - Aspartate aminotransferase, ALT - Alanine transaminase, A/G (Albumin/globulin) ratio.
